# Fully-Polymeric pH Sensor Realized by Means of a Single-Step Soft Embossing Technique

**DOI:** 10.3390/s17051169

**Published:** 2017-05-20

**Authors:** Paola Fanzio, Chi-Tung Chang, Maciej Skolimowski, Simone Tanzi, Luigi Sasso

**Affiliations:** 1 Department of Precision and Microsystems Engineering (PME), Delft University of Technology, 2628 CD Delft, The Netherlands; C.T.Chang@student.tudelft.nl (C.-T.C.); L.Sasso@tudelft.nl (L.S.); 2 Micronit Microtechnologies B.V., 7521 PV Enschede, The Netherlands; Maciej.Skolimowski@micronit.com (M.S.); Simone.Tanzi@micronit.com (S.T.)

**Keywords:** PEDOT, organic electrode, soft embossing, pH sensing

## Abstract

We present here an electrochemical sensor microsystem for the monitoring of pH. The all-polymeric device is comprised of a cyclic olefin copolymer substrate, a 200 nm-thin patterned layer of conductive polymer (PEDOT), and a 70 nm electropolymerized layer of a pH sensitive conductive polymer (polyaniline). The patterning of the fluidic (microfluidic channels) and conductive (wiring and electrodes) functional elements was achieved with a single soft PDMS mold via a single embossing step process. A post-processing treatment with ethylene glycol assured the functional enhancement of the electrodes, as demonstrated via an electrical and electrochemical characterization. A surface modification of the electrodes was carried out, based on voltammetric electropolymerization, to obtain a thin layer of polyaniline. The mechanism for pH sensing is based on the redox reactions of the polyaniline layer caused by protonation. The sensing performance of the microsystem was finally validated by monitoring its potentiometric response upon exposure to a relevant range of pH.

## 1. Introduction

pH is a key indicator for many biochemical processes and its monitoring is an essential process for many applications in the food and cosmetic industries, for environmental and agricultural monitoring and for healthcare. In particular, the need for miniaturized pH sensors has grown constantly over the past years, pushed by a great interest in the realization of complex Lab-on-a-chip and wearable devices able to continuously track specific health indicators [1,2], for instance for perspiration analysis [3,4], diabetes monitoring [5], and wound healing [6,7].

In this context, the development of a wearable sensor manufactured in fully-polymeric materials has several advantages compared to those created via conventional Si-based microfabrication [8]. Aside from exploiting the many well characterized, simple, and low cost techniques that can be employed in polymer micro-manufacturing, the use of solely polymeric materials increases the opportunities for functional integration. Developing micro-devices composed of similar polymeric materials also allows for the translation of complex cleanroom fabrication processes into mold-based replication techniques like embossing and imprinting. There is a broad range of suitable materials that can be employed in these fabrication approaches, like polydimethylsiloxane (PDMS) and cyclic olefin copolymers (from TOPAS Advanced Polymers GmbH, Frankfurt-Höchst, Germany), particularly for their biocompatibility, transparency, and mechanical properties [9]—key features that make them excellent materials for flexible microfluidic devices.

With the relatively recent development of conductive plastics, polymers can be used not only as substrates or as structures for fluidic handling, but also for the fabrication of organic electrodes [10,11,12]. The most widely used conductive polymer is poly (3,4-ethylenedioxythiophene) (PEDOT) thanks to its high conductivity, transparency, and environmental stability [13]. In particular, its conductivity and its solubility can be tuned with the addition of dopants such as the polyelectrolyte poly (styrenesulfonic acid) (PSS) [14] which makes it water soluble to ease the fabrication process, isopropanol (IPA) [15] to allow to increase the adhesion for spin-coating, ethylene glycol (EG) [16] to increase the conductivity and decrease the water solubility post-processing, organic solvents (like dimethyl sulfoxide) [17], acids (like H_3_PO_4_) [18], and carbon nanotubes [19] to increase the conductivity.

However, the micro-scale manufacturing of PEDOT is a major challenge in the implementation of this material in functional devices, particularly in the coupling and alignment of microfluidic channels and electrodes. In fact, patterning PEDOT with standard lithographic techniques is complex and requires the use of multi-step processes and/or specific dopants [20], and other techniques such as in situ electropolymerization, inkjet-printing techniques, lithography assisted chemical polymerization, and laser ablation suffer from several drawbacks, such as long fabrication times and high costs [21]. Moreover, PEDOT is not a thermoplastic polymer and standard thermoforming techniques like hot embossing cannot be used directly on this material [22]. An interesting approach is to use hot embossing to transfer a desired pattern on a thin PEDOT film spin-coated on a sacrificial thermoplastic polymer layer. This technique is called intermediate layer lithography [23] and has several advantages. It has been demonstrated by Kafka et al. [24] that it is possible to fabricate in a single hot embossing step both microfluidic and electrode functionalities, improving the fabrication throughput and reducing the problem of alignment. We recently validated that this integrated approach for polymer microfabrication is compatible with soft embossing (in which a polymeric mold is used instead of a hard one, made for instance of metal or silicon), as well as with a higher resolution than previously reported (1 μm) [25]. This technique opens up possibilities in tuning device functionality via mold prototyping and reduces the problem of mold breaking and costs. 

This approach is interesting for the realization of a sensing platform that can be subsequently locally activated specifically, e.g., for pH sensing. Since PEDOT is not intrinsically pH sensitive, it is necessary to chemically modify the electrode with a suitable pH-responsive layer. Several polymers exhibit potentiometric responses depending on the pH changes due to the presence of amino groups available for protonation. Electrochemical techniques are ideally suited for the in situ deposition of such polymers on the surface on the PEDOT electrodes [1]. Indeed, electrosynthesis allows the local deposition of polymers with a strong bond to the surface of the electrode. The most common pH sensitive polymers are polyaniline (PANI), some polyethylenimine derivatives (such as linear Polyethylenimine L-PEI), and polypyrrole (PPy) [26]. PEI and PPy are not stable for long periods but their biocompatibility has been proven. PANI is the more sensitive polymer for pH sensing but its biocompatibility is still under debate [27].

This paper describes an all-polymer microsystem comprised of a microfluidic channel with two integrated polymeric electrodes (made of PEDOT), fabricated by means of a single soft embossing step. The fabrication process performance and the conductive functionality of the electrodes have been characterized by means of four-point probe measurements and cyclic voltammetry. Furthermore, we have performed a post-treatment with ethylene glycol that allows an increase of PEDOT conductivity and a decrease of its water-solubility, which is an unwanted feature in biosensing devices. The electrochemical deposition of a PANI layer on the top of an indicator electrode gives the pH sensitivity to the device. This pH sensitive layer has been characterized via scanning electron microscopy and electrochemical techniques. Finally, proof-of-concept potentiometric measurements demonstrate that the microsystem is responsive to a range of pH changes and can therefore be applied to pH monitoring in wearable biosensing applications.

## 2. Materials and Methods

**Figure 1:**
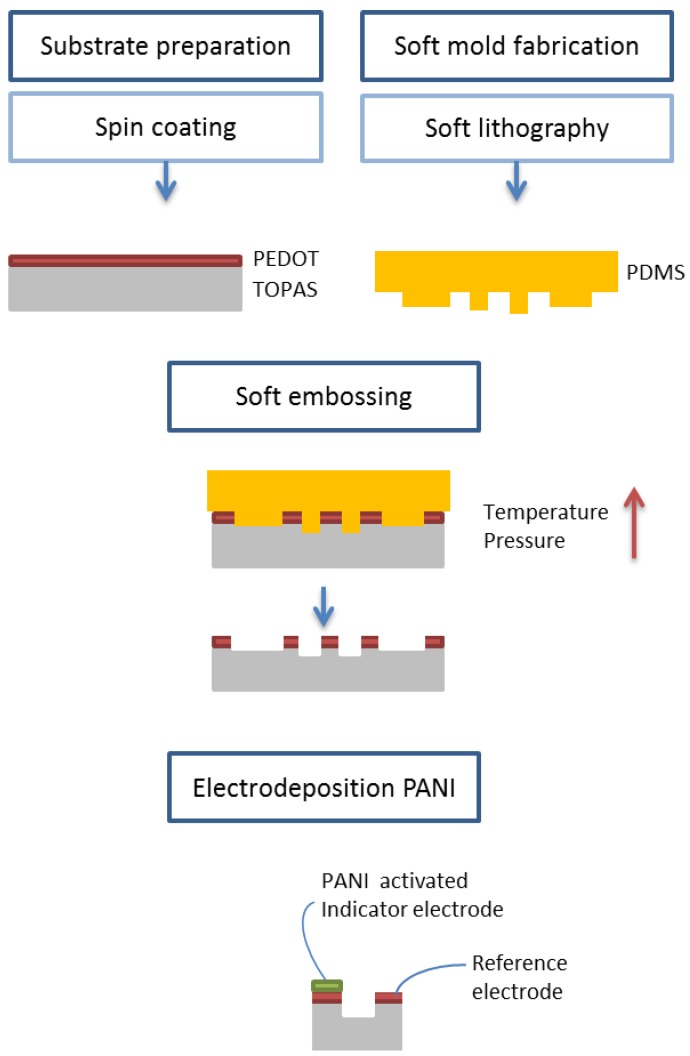
Scheme of the fabrication process.

Figure 1 shows a schematic diagram of the fabrication process, which can be divided into several steps. First, the substrate is prepared and the mold is fabricated by soft lithography. Then, the embossing process is carried out by placing the mold and the substrate in contact. Increasing the temperature and applying a pressure the pattern, present on the mold, is transferred to the substrate. Finally, it is necessary to activate the device, making it pH sensitive by electrodeposition of a polyaniline layer.

The master molds (in polystyrene or aluminum) were created via micromilling with a Minitech Minimill 3 machine (Minitech Machinery Corporation, Norcross, GA, USA). For the milling process, a 1.0 mm, two flute square end mill (Kyocera Micro Tools, Hendersonville, NC, USA) was used. The feed rate and spindle speed were 300 mm/min (XYZ) and 25,000 RPM, respectively. 

**Figure 2:**
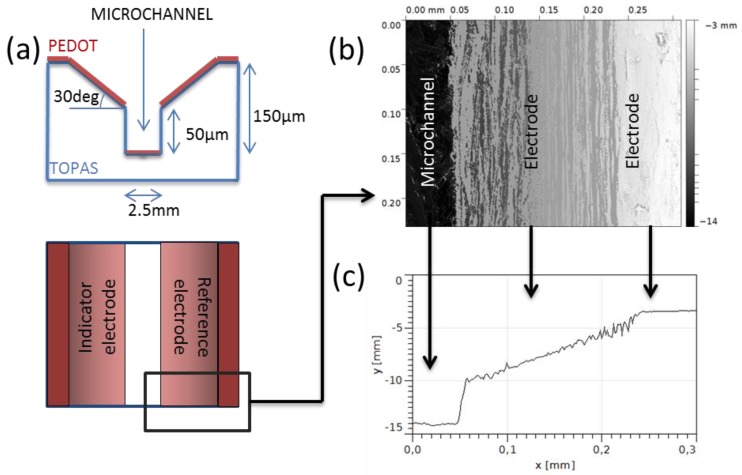
(**a**) Design of the device. A microfluidic channel is integrated with two PEDOT electrodes with oblique sidewalls (in red); (**b**) Top-view optical image of the device embossed on a PEDOT/TOPAS substrate; (**c**) Height profile of the embossed microchannel.

Microchannels (1 cm long, 2.5 mm wide, and 150 μm deep) were designed with sloped sidewalls (angle = 30°) in order to create two facing electrodes (indicator and reference electrodes), as shown in Figure 2a. Several geometries have been tested in order to evaluate the channel height and the sidewall angle (see supplementary information).

The soft working stamp, made of polydimethylsiloxane (PDMS, elastomer curing agent ratio 10:1), was prepared by first pouring PDMS on a polystyrene master mold and, after degassing for 1 h, by curing in an oven at 40 °C for 12 h. After peeling off the cured soft working stamp, it was baked in oven at 170 °C for 3 h in order to finish the curing and to increase the stiffness of the material.

A cyclic olefin copolymer (COC) wafer from TOPAS Advanced Polymers GmbH,Frankfurt-Höchst, Germany (grade 6013, a thermoplastic polymer with glass transition temperature equal to 136 °C) was rinsed with isopropanol and cleaned in oxygen plasma (2 min at 60 W). Poly(3,4-ethylenedioxythiophene) polystyrene sulfonate (PEDOT: PSS 1.3 wt% dispersion in water from Sigma Aldrich, St. Louis, MI, USA) (PEDOT: PSS) was mixed with isopropanol (various concentrations) and then spin-coated on the COC substrate (1000 rpm, 30 s, 500 rpm/s) and immediately dried at 50 °C for 5 min. The resulting thickness of the PEDOT layer was measured with a profilometer and it is equal to (116 ± 4) nm.

The sheet resistance was evaluated by means of four-point probe measurements (Signatone couple with a Keithley Sourcemeter 2400 and Nanovoltmeter 2182, Cleveland, OH, USA).

The embossing process was performed with an EVG embosser, with software-controlled pressure (6 kN, gradient 1 kN/min) and temperature (185 °C) for 9 min. The pressure was applied during cooling until the temperature reached 100 °C.

The soft embossing process has been compared with standard hot embossing performed with an aluminum mold, fabricated via micromilling, with the same geometries of the PDMS mold (the negative of the geometries in the polystyrene mold). In this case, the embossing was performed at 170 °C for 11 min with an applied force of 8 kN (gradient 1 kN/min).

For the characterization of the fabrication process, 3D optical images of the structures before and after the embossing were acquired with a white-light interferometer (Bruker). All the electrochemical experiments were carried out with a Potentiostat (Metrohm Autolab B.V., Utrecht, The Netherlands).

The embossed device was immersed in ethylene glycol (Sigma-Aldrich, St. Louis, MI, USA) for 10 min, then rinsed with water and dried with compressed air.

The polyaniline layer was deposited on the PEDOT indicator electrode by cyclic voltammetry. A droplet (50 μL) of a solution containing 0.05 M aniline (Sigma Aldrich, St. Louis, MI, USA) and 0.1 M KCl was placed in the microfluidic channel, and the electropolymerization was carried out by applying 80 cycles of potential scans (−0.5 V to 0.9 V, at 100 mV/s). The use of a KCl solution compared to a more conventional acidic solution makes this process more appealing for industrial settings. 

The pH detection experiment was carried out in buffers with different pH values (Hanna Instruments, Nieuwegein, Netherlands) and by measuring the open circuit potential (OCP). 

## 3. Results

### 3.1. Substrate Preparation

**Figure 3:**
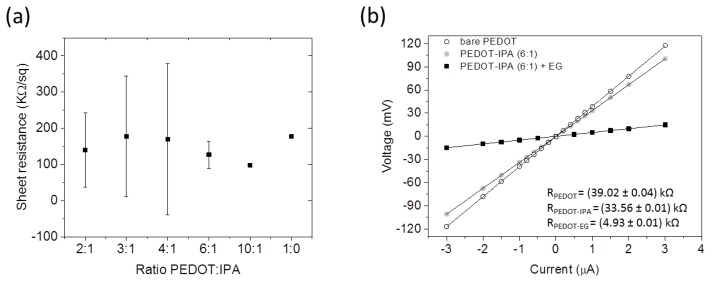
(**a**) Effect of PEDOT:IPA solution concentration on obtained film resistance (error bars represent one standard deviation on 4 measurements); (**b**) Voltage/Current curves measured on a spin-coated film (1000 rpm, 30 s, 500 rpm/s) made of PEDOT: PSS (empty circles), PEDOT-IPA 6:1 (grey circles) and after the treatment with ethylene glycol (black square). Lines correspond to the linear fit for each curve.

The first step of the fabrication process is the realization of the substrate made of a thin film of PEDOT spin-coated on a COC wafer substrate. However, the spin-coating process is affected by the poor wettability of the COC surface, resulting in a low adhesion between PEDOT and COC (the measured contact angle with water for the COC substrate is about 81°). This leads to the creation of inhomogeneous coverage of the COC substrate. In order to increase the homogeneity of the spin-coated thin film, isopropanol (IPA) was added to PEDOT. After mixing, the solution was spin-coated and the sheet resistance was measured with a four-point probe. Figure 3a shows the effect of IPA concentration in the spin-coating solution on the sheet resistance of the obtained film. Solutions with low IPA concentrations (PEDOT:IPA 10:1 in volume) resulted in more conductive films, with lower sheet resistances, and also in high film homogeneity and complete coverage of the COC wafer. On the other hand, increasing the IPA concentration too much (PEDOT:IPA 4:1, 3:1, 2:1) does not significantly affect the PEDOT conductivity but decreases the film homogeneity and therefore yields higher fluctuation in the values for the sheet resistance measured in different substrates and even on the same substrate. This inhomogeneity is due to the high dilution of the PEDOT [15] which results in a non-uniform presence of the conductive polymer in the thin film. We used a PEDOT:IPA 6:1 ratio which gives the best uniformity of the layer both in terms of homogeneity and conductivity.

After baking the spin-coated PEDOT layer, a post-treatment has been performed in order to increase the conductivity of the PEDOT film and decrease its water solubility. Indeed, it has been demonstrated that a treatment with ethylene glycol (EG) produces a change in the PEDOT conformation structure from a coiled conformation (benzoid structure) to a more linear one (quinoid structure) that is responsible for the increased change transfer in the film and the increased stability of PEDOT in contact with water-based solutions [16]. The enhancement in the conductivity has been measured with a four-point probe setup. The result is shown in Figure 3b where the voltage/current plot obtained on a PEDOT film treated with EG (black squares) is compared with the ones obtained on a PEDOT-IPA (grey dots) and PEDOT (empty dots) films. The electrical resistance has been evaluated by means of a linear fit of the curves. We have found that the EG treatment produces a decrease in the resistance of about eight times from about 40 kΩ to about 5 kΩ. This result is even more important since it has been demonstrated that the high temperature during the embossing process could decrease the PEDOT conductivity due to the presence of PSS molecules that increase the PEDOT hygroscopicity [28]. 

In conclusion, the substrate preparation was optimized in order to obtain a thin PEDOT layer on a thermoplastic COC substrate with high conductivity, homogeneity, and stability in water.

### 3.2. Embossing Process

We designed devices with the geometry shown in Figure 2a. The sidewalls of the microchannel are oblique in order to create two electrodes in contact with the solution. We patterned our device design in the previously mentioned conductive layer for the realization of organic electrodes integrated in a microchannel via a soft embossing process. The soft embossing is mainly a hot embossing process in which a polymeric mold is used instead a hard one. PDMS was chosen as the material for the soft mold due to its easy manufacturability and broad use.

It is important to underline that PEDOT is not a thermoplastic polymer and, in principle, it cannot be imprinted. However, a thin layer of PEDOT on top of a thermoplastic polymer can be indirectly patterned via embossing. In fact, the pattern is transferred from the mold to the COC and the PEDOT layer follows the pattern by deforming its structure and, eventually, if the height and geometry of the embossed structure is carefully chosen, it breaks. These broken edges allow the creation of decoupled electrodes, as shown in Figure 2a. The sidewall slope can furthermore be tuned to avoid damaging the electrode in the geometrical bend. We have evaluated the decoupling of the electrodes in a function of the channel geometry (see supplementary information) and we have found that the best channel geometry consists of a rectangular microchannel, at least 50 μm deep, with sloped side walls with an angle of 30°, as shown in Figure 2a.

Figure 2b,c shows, respectively, a top-view optical image of the device and the height profiles. The fidelity of the pattern is excellent, especially in the vertical channel side walls, which must be sharp enough to cut the PEDOT layer. The decoupling of the two electrodes was evaluated in 63% of the fabricated devices. 

We have also compared the results of the soft embossing with a standard hot embossing process performed with an aluminum mold. We have evaluated that the decoupling and the fidelity of the pattern after the embossing process is the same with both fabrication techniques. The difference is the fact that, for the soft embossing, it is necessary to increase the temperature and decrease the pressure during the embossing. This is due to the fact that the PDMS mold is a soft material and, in order to reduce pattern deformations, it is necessary to increase the viscosity of the COC (increase the temperature) and decrease the mechanical stress on the mold (decrease the pressure). 

The use of a polymeric mold has several advantages compared with the use of hard one. Indeed, occasional mold breaking is reduced compared to embossing with Si molds, and the fabrication of the soft working stamp is cheaper compared with the fabrication of Ni-plated molds, based on a soft lithography process. Micro-milling is also a cheap technique for mold fabrication but the range of structures geometries and dimensions that can be fabricated is not as broad as for soft lithography. Moreover, soft molds can be used to reduce thermal expansion coefficient mismatches with the substrate and they can be used several times, decreasing the fabrication costs. On the other hand, standard hot embossing processes require lower temperatures and the molds are less prone to aging. In this respect, we have found that PDMS molds can be used for a maximum of four times before starting to degrade, compromising the results of the embossing, i.e., the realization of decoupled electrodes. 

Integrating the creation of both electrode and fluidic functionalities in a single fabrication step is attractive for the reduction of manufacturing costs and therefore to increase the manufacturability of the device.

### 3.3. Surface Modification

In order to make the device pH sensitive, a polyaniline (PANI) layer was electrochemically deposited on the indicator electrode via cyclic voltammetry.

It is well known that PANI exists in three different base forms: leucoemeraldine (LEB, fully reduced), emeraldine (EB, semi-oxidized), and pernigraniline (PNB, fully oxidized). The EB form of PANI can be reversibly protonated with sufficiently strong acids to its emeraldine salt form (ES) due to the presence of basic sites (amine and imine groups) in the polymer structure. ES is the protonated form of EB and it is the only electrically conducting form of PANI. The pH sensitivity of PANI is based on the equilibrium between EB and ES [29].

**Figure 4:**
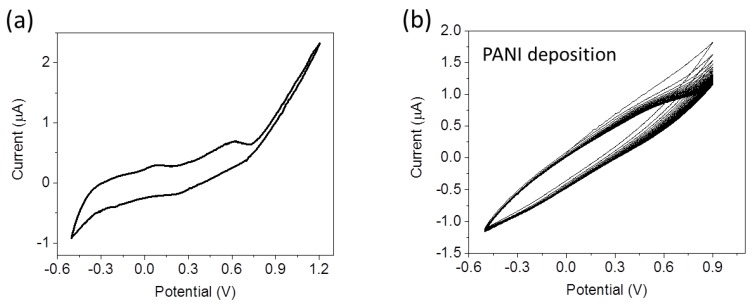
(**a**) Cyclic voltammetry measured in the aniline solution (0.05 M aniline + 0.1 M KCl) recorded at a scan rate equal to 10 mV/s; (**b**) PANI deposition by 80 CV cycles at 100 mV/s scan rate.

Figure 4a shows a cyclic voltammogram recorded in the aniline solution with a scan rate equal to 10 mV/s during electropolymerization. Two peaks are present at 0.08 V and 0.64 V corresponding to the two oxidation states of PANI. The first redox couple at V = 0.08 V corresponds to the transition between LEB and ES, the second redox couple at V = 0.64 V corresponds to the transition between ES and PNB [29].

The PANI deposition has been carried out during 80 cycles performed between −0.5 V and 0.9 V at 100 mV/s scan rate. The results of this deposition process yield voltammograms as shown in Figure 4b. The oxidation currents progressively increase with time reflecting the growth of the conductive PANI layer. The thickness of the PANI layer was calculated by measurements of the voltammetric accumulated charge during the electropolymerization [27] and it corresponds to about 70 nm.

**Figure 5:**
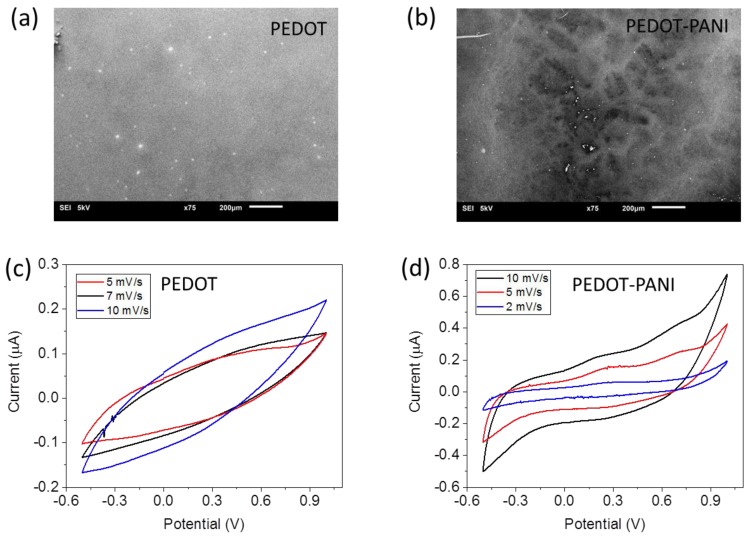
SEM images of the indicator electrode surface: (**a**) before the PANI deposition; (**b**) after the PANI deposition. CV curves at different scan rates in KCl 0.1 M (**c**) before the PANI deposition; (**d**) after the PANI deposition.

The surface morphology of the electrodes was evaluated by SEM imaging. The surface of the PEDOT electrode of Figure 5a is relatively smooth and uniform. On the contrary, the surface of the modified PEDOT-PANI electrode of Figure 5b is of higher roughness, demonstrating that the PANI layer has been effectively grown on the PEDOT indicator electrode.

Finally, we have compared the electrochemical properties of the PEDOT and PEDOT-PANI indicator electrodes by cyclic voltammetry (CV). Figure 5c,d shows the cyclic voltammograms at different scan rates obtained in KCl 0.1 M respectively before and after the PANI deposition. Despite the absence of clear peaks, the response of PEDOT demonstrates that the electrodes are electrochemically active. The CVs of PEDOT-PANI show an increased electrochemical response with the appearance of two redox peaks, corresponding to the two oxidation states of PANI described above. We do not observe a significant peak shift upon increasing the scan rate. The current peak amplitude linearly increases in with varying scan rates demonstrating a pseudo-reversible electrode behavior for PEDOT-PANI electrodes.

### 3.4. pH Sensing Experiment

**Figure 6:**
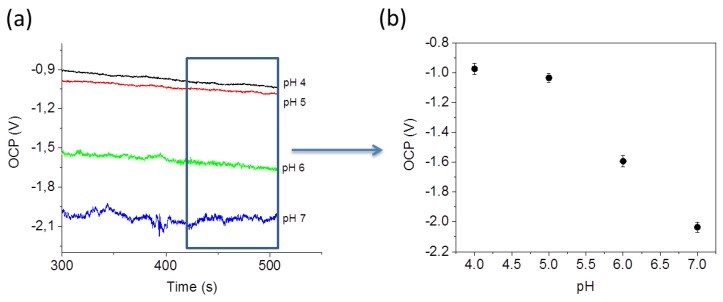
(**a**) Open current potential (OCP) recorded at different pH values in function of the time; (**b**) Mean values for the OCP at each pH value (error bars represent the standard deviation).

The pH sensing experiment was carried out by measuring the open circuit potential (OCP). OCP traces, shown in Figure 6a, have been recorded by placing different pH buffer solutions in the microfluidic channel. The PANI coated device exhibits a high pH sensitivity in the range between 5 and 7 pH. The equilibrium is reached after about 100 s (see supplementary information). Figure 6b shows the mean values of the OCP calculated after reaching the equilibrium (between 300 s and 500 s) for each pH value. 

Another aspect that is necessary to take into account is the stability of electrodes. Indeed, we have observed that the pH measurement recording can be performed for maximum of 1 h. After this period, the fluctuation in the measured OCP increases.

The results of this last section of the work demonstrate that the concept of our all-polymeric pH micro-sensor is suitable for the monitoring of these bio-parameters in wearable devices. Even before the careful calibration and engineering optimization typically carried out before a device of this sort moves into a higher technology-readiness level, our system demonstrates an ability to detect pH in the health-relevant range almost linearly. Furthermore, the fact that the measurement is performed by keeping the current fixed at zero and recording the potential of the indicator electrode makes the device easily integrable into more complex platforms without the need of additional in situ electronics. Measurements could be performed by connecting an external device and by simply recording the potential. 

## 4. Discussion

We have developed a microfluidic device with integrated polymeric electrodes by means of a single-step soft embossing process. This result is based on the synergetic process integration between soft embossing (a cheap and high throughput polymer micro-fabrication technique), intermediate layer lithography (for patterning conductive polymers), and an optimized micro-channel geometry. Our devices consist of three polymer layers: a COC as substrate, a patterned PEDOT layer as functional electrodes and wiring, and an electropolymerized PANI layer for the pH sensing capability. 

We have optimized the uniformity and conductivity of the conductive layer by adding IPA to the PEDOT solution and a post-treatment with EG. These are critical aspects for the realization of PEDOT electrodes: in particular, the possibility of increasing the conductivity and decreasing the water solubility has a great impact on the use of this polymer to create flexible wiring for wearable devices.

Moreover, we have demonstrated that the described microsystem functions as a pH sensor via potentiometric measurement of the redox PANI reactions upon exposure to different pHs. Although our developed system operates as a proof-of-concept, we envision the implementation of both our technology as well as our biosensing system design into more complex bioanalytical platforms.

## Supplementary Materials

The following are available online at http://www.mdpi.com/1424-8220/17/5/1169/s1, Figure S1: Scheme of the device and picture of the experimental setup; Figure S2: Comparison between the percentage of coupled and decoupled electrodes for hot embossing (HE) and soft embossing (SE) in different geometries; Figure S3: Parameters using for the embossing; Figure S4: 3D optical images of the used molds and the embossed substrate; Figure S5: Open current potential (OCP) values recorded at pH 6 in function of the time.
