# Supplementary material for: Fully-Polymeric pH Sensor Realized by Means of a Single-Step Soft Embossing Technique
Source: Sensors (Basel). 2017 May 20;17(5):1169. doi: 10.3390/s17051169 (PMC5470914; doi:10.3390/s17051169)
Supplement: Supplementary file 1 [file sensors-17-01169-s001.pdf]

Supplementary Information

1. Optimization of the Device Geometry

Several test structures have been evaluated in order to optimize the device geometry. There are two important parameters that must be taken into account (**Figure S1**):

1) The height of the channel which is related with the electrical decoupling of the electrodes;
2) The slope of the electrode which must be chosen in order to avoid cracking the PEDOT electrodes.

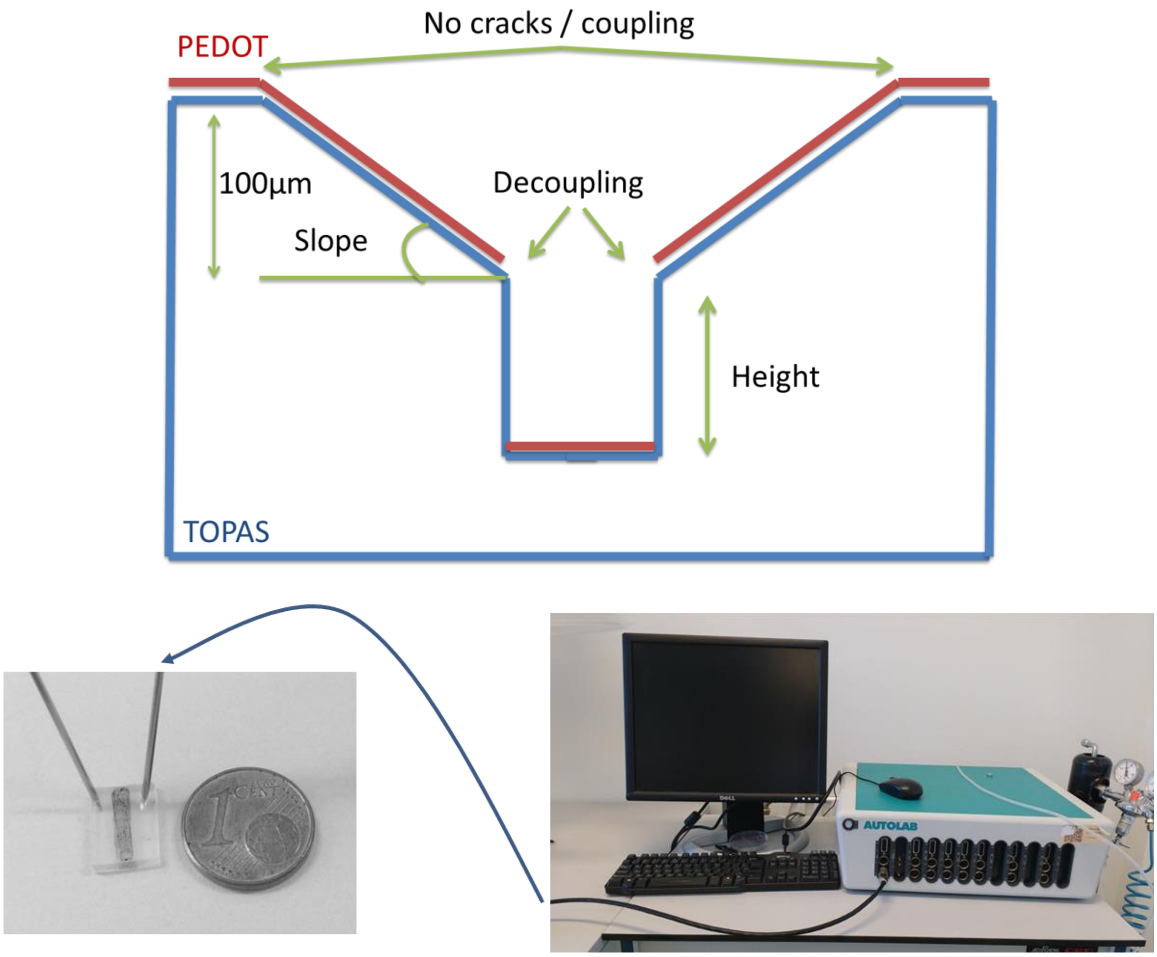

Figure S1. Scheme of the device and picture of the experimental setup.

The decoupling of the electrodes has been evaluated with a multimeter by placing the one probe inside the channel and the other one on each electrodes.

Figure S2 shows three tables in which the percentage of coupled and decoupled electrodes for hot embossing (HE) and soft embossing (SE) are compared in different geometries. Figure S2a shows the result obtained using rectangular cross section channels with different heights. For both HE and SE we have found that the height of the channel must be at least 50 µm in order to obtain electrically decoupled electrodes. Moreover, in order to avoid the formation of cracks in the electrodes in correspondence of the point where the electrodes are bent that could affect their conductivity, we have tested structures without the channel (Figure S2b). In this case, we have evaluated the percentage of coupled electrode, whose conductivity was not affected by the bending. The result is that the slope must be 30° or lower.

Finally, we have fabricated structures with both electrodes and channel (Figure S2c). Both the devices with height of 50 µm and 100 µm resulted decoupled. However, considering that we have

evaluated that the slope of the electrode must be lower than 30°, we have decided to use the first type of device, underlined in green (height 50 μm, slope 30°).

(a)

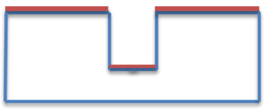

Height (μm)	% Decoupled HE	% Decoupled SE	
20	29	6	x
50	79	25	ok
100	75	81	ok

(b)

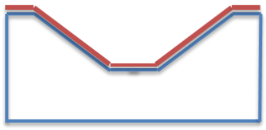

Angle (deg)	% Decoupled HE	% Decoupled SE	
10	0	13	ok
30	4	13	ok
60	63	56	x

(c)

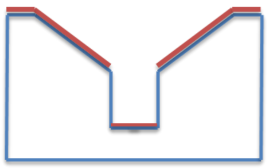

Angle (deg)	Height (μm)	% Decoupled HE	% Decoupled SE	
30	50	75	63	ok
10	20	8	0	x
60	100	100	88	x

Figure S2. Comparison between the percentage of coupled and decoupled electrodes for hot embossing (HE) and soft embossing (SE) in different geometries: (**a**) only the microchannel, (**b**) only electrodes and (**c**) microchannel and electrodes.

We have also found out that the result in terms of fidelity and reproducibility of the pattern obtained with the soft embossing is comparable with the one obtained with the hot embossing. Figure S3 shows the parameters used in the two fabrication processes.

	Force (N)	Temperature (◦C)	Time (min)	Force gradient (N/min)
Hot embossing	8000	170	11	1000
Soft embossing	6000	185	9	1000

Figure S3. Parameters using for the embossing.

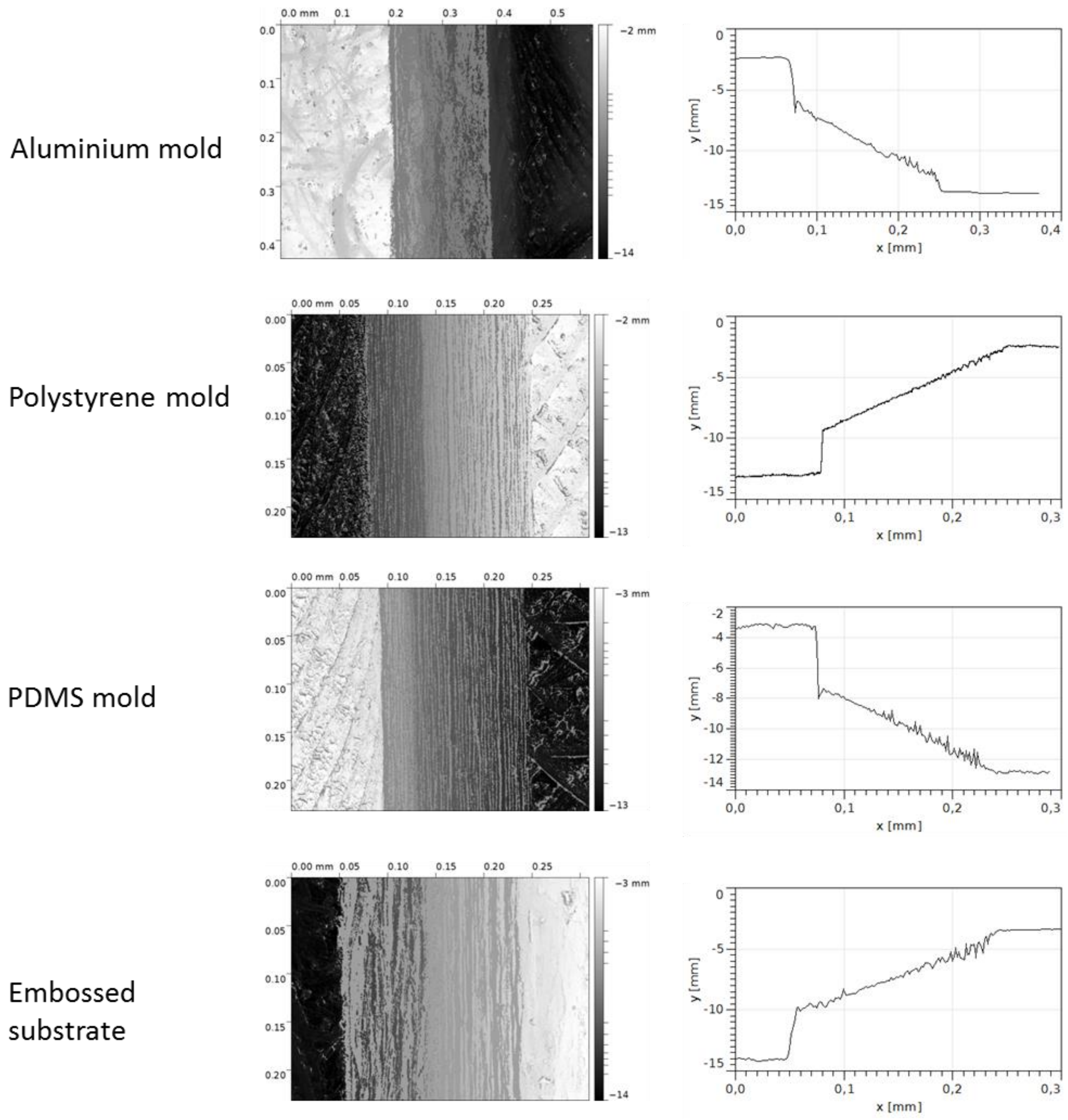

Figure S4. 3D optical images of the used molds and the embossed substrate.

2. Sensor Stabilization

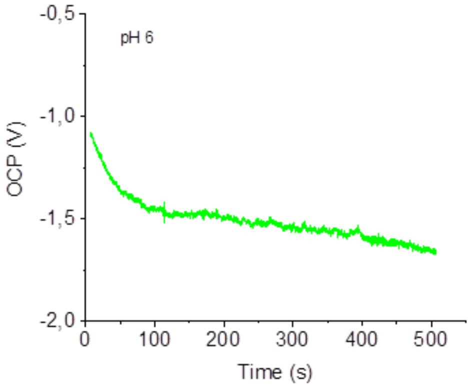

Figure S5. Open current potential (OCP) values recorded at pH 6 in function of the time.
